# Decoding Waldenström Macroglobulinemia Through Genomics, Epigenomics and Cellular Interactions

**DOI:** 10.3390/ijms27125173

**Published:** 2026-06-07

**Authors:** Tereza Růžičková, Michal Kaščák, Zuzana Chyra, Viera Sandecká, Jana Kotašková, Sabina Ševčíková

**Affiliations:** 1 Babak Myeloma Group, Department of Pathophysiology, Faculty of Medicine, Masaryk University, 625 00 Brno, Czech Republic; 2 Department of Internal Medicine, Hematology and Oncology, University Hospital Brno, 625 00 Brno, Czech Republic; 3 Department of Hematooncology, University Hospital Ostrava, 708 00 Ostrava, Czech Republic; 4 Department of Hematooncology, Faculty of Medicine, University of Ostrava, 702 00 Ostrava, Czech Republic; 5 Institute of Medical Genetics and Genomics, University Hospital Brno, Faculty of Medicine, Masaryk University, 625 00 Brno, Czech Republic

**Keywords:** Waldenström macroglobulinemia, *MYD88*, *L265P*, *L252P*, *CXCR4*, *TP53*, microRNA, signaling pathways

## Abstract

Waldenström macroglobulinemia is a rare lymphoplasmacytic malignancy characterized by bone marrow infiltration and monoclonal immunoglobulin M production. Despite its indolent clinical course, the disease exhibits considerable biological complexity driven by recurrent genetic alterations, dysregulated signaling pathways, and a supportive bone marrow microenvironment. The identification of *MYD88* and *CXCR4* mutations has significantly advanced understanding of disease pathogenesis, clonal evolution, and treatment response. Additional insights from cytogenetic, epigenetic, and microRNA studies have further refined the molecular landscape of this disorder. These advances have translated into improved therapeutic strategies, particularly with the introduction of Bruton’s tyrosine kinase inhibitors, although treatment resistance remains a clinical challenge. Other targeted approaches, including BCL2 and proteasome inhibition, offer additional options for personalized therapy. Ongoing integration of molecular and microenvironmental data is expected to enhance risk stratification and support the development of more effective, tailored treatment strategies.

## 1. Introduction

Waldenström macroglobulinemia (WM) or in other words immunoglobulin M (IgM) lymphoplasmacytic lymphoma (LPL) was first described by J. G. Waldenström in 1944 [1]. It is a rare, indolent B-cell malignancy, with an age-adjusted incidence of approximately 3.6 per 1,000,000 [2,3]. It is characterized by clonal expansion of lymphoplasmacytic cells, plasma cells (PCs), and lymphocytes within the bone marrow (BM), accompanied by the overproduction of monoclonal IgM [4,5,6]. These features differentiate WM from other types of LPL called non-WM type LPL that include IgG or IgA LPL, non-secretory LPL, and IgM LPL without BM involvement [7]. WM is classified as a distinct clinical entity within the spectrum of non-Hodgkin lymphomas and shares overlapping features with both multiple myeloma (MM) and chronic lymphocytic leukemia (CLL) [8,9,10].

The median age at diagnosis is 70 years, and the median overall survival (OS) is 8 years [2,11,12]. Given its typically indolent course, the most common causes of death among WM patients are often unrelated to the malignancy itself [13,14]. Consequently, treatment strategies for WM are primarily focused on disease control rather than curative intent [11].

The pathogenesis of WM is strongly driven by genetic alterations, with the *MYD88^L265P^* (myeloid differentiation primary response 88) mutation being the most prevalent, occurring in over 90% of WM cases [15]. This mutation leads to constitutive activation of the NF-κB (nuclear factor kappa light chain enhancer of activated B cells) signaling pathway, thereby promoting cell survival and proliferation [16]. In addition, mutations in *CXCR4* (C-X-C chemokine receptor 4) are observed in approximately 30–40% of patients and are associated with altered cell trafficking, contributing to resistance against BTK (Bruton’s tyrosine kinase) inhibitors [17,18,19].

Multiple intracellular signaling pathways, including NF-κB, BCR (B cell receptor), PI3K/Akt/mTOR (phosphatidylinositol 3-kinase/protein kinase B), JAK/STAT (Janus kinase/signal transducer and activator of transcription proteins), and CXCR4/SDF1 (stromal cell-derived factor 1), are dysregulated in WM, contributing to tumor cell survival, proliferation, and drug resistance [4,15,17,18]. The complexity of WM is not only shaped by genetic alterations but also by the interplay between malignant B cells and their BM microenvironment. The tumor niche provides essential survival signals through cytokines, chemokines, and extracellular vesicles, further activating key oncogenic pathways [20]. Targeting these pathways has revolutionized WM therapy, particularly with BTK inhibitors (BTKi), such as ibrutinib and zanubrutinib, which have shown remarkable efficacy in patients with *MYD88*-mutant disease [14,18]. Nevertheless, resistance mechanisms, partially mediated by alternative signaling pathways, remain a significant challenge, underscoring the need for a deeper understanding of the molecular landscape of WM.

## 2. Clinical Presentation

WM patients typically present with symptoms related to elevated serum IgM levels and BM infiltration by malignant cells. However, up to 25% of patients may be asymptomatic at the time of diagnosis and have comparable survival to the matched general population [21,22,23,24,25].

The most common clinical features of WM patients include B-symptoms, cytopenias—particularly anemia—which may result from impaired erythropoiesis due to BM infiltration, iron deficiency inducing absolute and/or functional deficiency due to bleeding and/or hyperhepcidinemia (WM cells produce hepcidin), or infrequently hemolysis [11,26,27,28,29].

WM involvement may manifest itself as lymphadenopathy (in 15% of patients), splenomegaly (in 15% of patients), or hepatomegaly (in 20% of patients), and rarely Bing–Neel syndrome (in 1% of patients), in which the central nervous system is infiltrated by lymphoplasmacytic cells [30,31,32,33]. Extramedullary disease occurs in approximately 20–30% of WM patients, mainly in the nervous system, kidneys, or lungs [34,35]. Non-specific symptoms accompanying this disease include the so-called B symptoms: pathological fatigue, as well as weakness, nausea, excessive sweating, fever, or weight loss [30,36].

Elevated serum IgM levels (typically >4000 mg/dL) can induce hyperviscosity syndrome historically reported in up to one-third of patients [37]. This life-threatening condition results from the aggregation of IgM pentamers, which increases serum viscosity by more than twofold. Clinical manifestations include skin and mucosal bleeding, retinopathy, headache, confusion, and, in rare instances, cardiovascular complications [38,39].

Additionally, the presence of monoclonal IgM can lead to a range of systemic complications, including peripheral neuropathy, AL amyloidosis, cryoglobulinemia, and cold agglutinin disease [30,32,40,41,42]. WM-related amyloidosis is caused by the aggregation of poorly folded proteins (mostly Ig light chains), which deposit in tissues as insoluble fibrils, leading to organ dysfunction [43,44]. Cryoglobulinemia is characterized by precipitation of IgM at temperatures below 37 °C, whereas cold agglutinin disease arises from autoreactive IgM antibodies that agglutinate red blood cells, particularly in cooler body regions [31]. IgM-related polyneuropathy, most commonly IgM anti-myelin-associated glycoprotein (anti-MAG) paraproteinemic peripheral neuropathy, occurs in 13–22% of WM patients [45,46,47]. Anti-MAG peripheral neuropathy is a slowly progressive demyelinating neuropathy characterized by gait instability, balance impairment, and sensory deficits, ultimately leading to significant disability in nearly half of affected patients over the course of the disease [48].

Another potential complication that can occur in WM patients is Schnitzler syndrome, a rare autoinflammatory disorder characterized by chronic, recurrent urticaria [30,31,32]. The associated exanthema often affects the entire body [49,50]. The lesions are transient and generally resolve within 48 h. The rash tends to follow a distinct daily pattern, often worsening in the evening hours before gradually subsiding [50,51].

## 3. Pathogenesis

**Figure 1:**
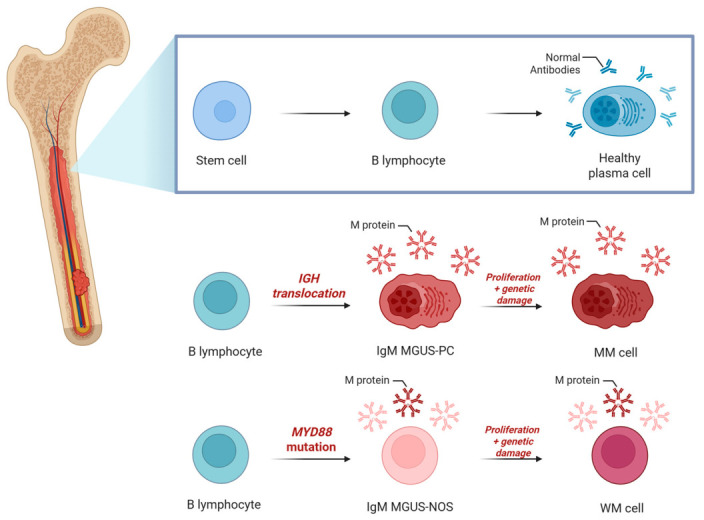
Normal plasma cell differentiation and contrasting evolutionary trajectories of IgM-secreting disorders. The upper panel illustrates physiological B cell maturation: from stem cell to naïve B lymphocyte and finally to a healthy plasma cell producing normal immunoglobulins. The lower panel depicts two pathological branches: (1) IgM MGUS-plasma-cell (IgM MGUS-PC), driven by *IGH* translocation events in early progenitors, may evolve to IgM multiple myeloma (MM) through ongoing proliferation and genomic instability; (2) IgM MGUS-not otherwise described (IgM MGUS-NOS), arising from mature B cells carrying the *MYD88* mutation, represents the precursor state of Waldenström macroglobulinemia (WM). Both processes are characterized by the production of IgM monoclonal protein (M protein) and clonal expansion. Created in BioRender. Smith, J. https://app.biorender.com/c-248457.

WM typically develops from a premalignant disorder known as monoclonal gammopathy of undetermined significance (MGUS) [52,53]. Two distinct subtypes of IgM MGUS have been described so far. IgM MGUS-not otherwise described (IgM MGUS-NOS) is considered the precursor to WM and frequently harbors *MYD88* mutations. In contrast, IgM MGUS-plasma cell (IgM MGUS-PC) is thought to precede the development of MM and is characterized by the absence of *MYD88* mutations, but often presents with cytogenetic translocations involving the *IGH* (Immunoglobulin heavy locus) gene, which are typical for MM (Figure 1) [12,54,55,56].

WM is driven by the malignant transformation of post-germinal center B-lymphocytes that have already encountered antigen but have not undergone isotype class switching. These cells, which resemble memory B cells with lymphoplasmacytoid morphology, produce IgM and are detectable at the MGUS stage [57,58]. Arbitrary values of BM infiltration have been set to 10% for WM diagnosis; however, it is no longer recommended to diagnose patients based on this value, as some symptomatic patients may present with lower disease burden [59].

Morphologically, WM cells can range from small lymphocytes with aggregated chromatin, bland nuclei, and thin cytoplasm to mature and differentiated PCs, which are usually present in low numbers and may contain Dutcher bodies [60,61,62,63]. These PCs are likely derived from the malignant lymphoplasmacytic population and are considered the primary source of monoclonal IgM [63].

The immunophenotype of WM cells is variable, which may complicate differential diagnosis. Typically, WM cells express surface IgM with either κ or λ light-chain restriction, together with pan-B-cell markers such as CD19, CD20, CD22, and CD25. However, a subset of cases may also show expression of CD5, CD23, or CD10, markers that can overlap with other B-cell malignancies. Additional immunophenotypic features include variable expression of CD10 and CD23, positivity for CD27, CD52, and CD79, and negativity for CD11c and CD103. In addition, the PC component may express CD38 and CD138 [60,61,64,65,66,67,68,69].

Recent molecular studies of IgM monoclonal gammopathies have identified three distinct transcriptional, epigenetic, and immunological clusters of patients (Table 1) [70,71]. Although these clusters and the memory B cell-like (MBC-like)/plasma cell-like (PC-like) epigenetic subtypes described below both reflect biological heterogeneity within IgM monoclonal gammopathies, they should not be considered directly interchangeable classifications. The cluster-based model integrates transcriptional, epigenetic, metabolic, and immune features across IgM MGUS and WM, whereas the MBC-like and PC-like subtypes are primarily defined by DNA methylation patterns in *MYD88*-mutated WM.

**Figure 2:**
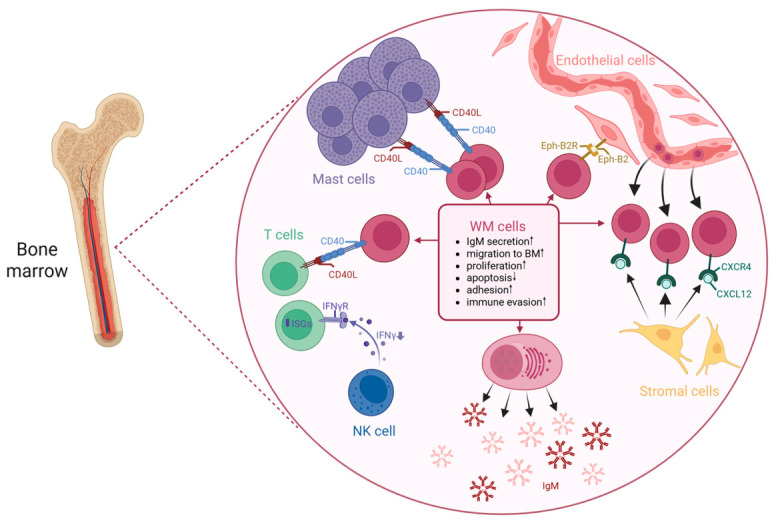
Cellular and molecular interactions shaping the Waldenström macroglobulinemia (WM)-supportive bone marrow (BM) microenvironment. The BM microenvironment provides multiple pro-tumorigenic signals to WM cells. CD40/CD40L engagement from mast cells and T cells promotes proliferation and IgM synthesis. Endothelial and stromal cells regulate trafficking and retention through CXCL12-CXCR4 and Ephrin-EphR signaling. Impaired NK cell function and reduced interferon γ (IFNγ) responsiveness enable immune evasion. These combined interactions drive hallmark features of WM biology, including enhanced IgM secretion, increased proliferation and adhesion, bone marrow tropism, and resistance to apoptosis. ISGs, interferon-stimulated genes. Created in BioRender. Smith, J. https://app.biorender.com/c-248457.

The BM microenvironment (Figure 2) plays a crucial role in WM pathogenesis [54]. In fact, the crosstalk between malignant cells and stromal or immune cells in the BM microenvironment favors disease progression and promotes IgM secretion [72]. Homing to the BM is a key characteristic of WM cells due to high expression of CXCL12 (C-X-C chemokine ligand 12) in the BM, which causes migration of WM cells [3]. Overproduction of mast cells in the BM is a typical histological finding in WM patients [73]. High mast cell density in BM was shown to be associated with increased tumor burden in the BM, several aggressive disease features, and poor clinical outcomes [74]. Mast cells promote WM cell proliferation, survival, and IgM synthesis mainly through CD40L-CD40 interaction [75]. CD40L-CD40 interaction between WM cells and Tregs was also observed. Following this interaction, Treg number increases, leading to immunosuppression and increased self-tolerance [76,77,78]. Endothelial cells have been described to enhance proliferation and adhesion of WM cells through the interaction of Ephrin receptor B2 (Eph-B2) expressed on WM cells and Eph-B2 ligand expressed on endothelial cells [79]. Natural killer cells in WM patients have been characterized by a decrease in interferon signaling, which is consistent with immune exhaustion and dysfunction of these cells in WM [80]. Similar downregulation of interferon-response genes has been observed in T cells, further contributing to immune evasion [80].

## 4. Genetic Architecture and Molecular Drivers

### 4.1. MYD88

Approximately 80–95% of WM patients harbor the somatic *MYD88* mutation historically referred to as *MYD88^L265P^* [15,18]. The reported prevalence of *MYD88* mutations in WM may partly depend on assay sensitivity, since very low-abundance *MYD88*-mutated subclones may remain undetected by less sensitive methods. The *MYD88* gene, located on chromosome 3p22.2, encodes a 296-amino-acid toll-like receptor (TLR) adaptor protein. The *MYD88^L265P^* mutation results in a substitution of leucine for proline at amino acid position 265 of the MYD88 protein. The cause of this substitution at the genetic level is a T>C substitution at position 794 of the *MYD88* transcript, originally numbered according to the transcript in use at the time of its discovery in 2011 [81]. Under current reporting standards, variants should be described using the MANE Select transcript (NM_002468.5), in which this mutation corresponds to L252P (c.755T>C) [82]. Despite this updated nomenclature, the historical designation “L265P” remains widely used throughout the literature.

In contrast, non-*L265P MYD88* mutations are rare and have been detected in only 1–2% of WM patients, including *S219C*, *M232T*, and *S243N* [18,83]. Structural alterations on chromosome 3p further contribute to the increased allele frequency of *MYD88* mutations in tumor cells. While deletions of the wild-type (WT) *MYD88* allele and amplifications of the mutant allele have been reported, the most prevalent alteration is acquired uniparental disomy, which results in a homozygous *MYD88*-mutated tumor genotype [84,85,86].

Interestingly, *MYD88* mutations have also been detected in early pre-B progenitor cells of WM patients, suggesting that the initiating oncogenic event may occur earlier in B cell development [80]. Supporting this theory, recent findings by García-Sanz et al. indicate that only clones harboring *MYD88^L265P^* can acquire additional genetic alterations that drive more aggressive disease phenotypes [87]. Several studies focusing on *MYD88*-mutated patients have identified two distinct biological subtypes: MBC-like and PC-like. The key distinguishing features between these groups are the higher prevalence of *CXCR4* mutations in the MBC-like subtype and del(6q) in the PC-like subtype [88,89].

Approximately 5–10% of WM patients do not carry *MYD88* mutations. These *MYD88WT* cases are characterized by a lower rate of BM infiltration and lower serum IgM levels [11,90]. However, they are associated with poorer OS [56,91].

The presence of the *MYD88^L265P^* mutation has both diagnostic and prognostic significance in WM. In cases where the histopathological diagnosis is uncertain but WM is suspected, the presence of the *MYD88* mutation strongly supports the diagnosis of WM [92]. Moreover, *MYD88^L265P^* serves as a predictive biomarker for therapeutic response, particularly to BTKi. In addition to flow cytometric detection of malignant clonal cells, droplet digital PCR (ddPCR) has been recently proven as a suitable tool for *MYD88^L265P^* screening and monitoring minimal residual disease (MRD) with a sensitivity of 5 × 10^−5^. Both unsorted BM and peripheral blood samples can be reliably tested, as well as circulating tumor DNA (ctDNA), which represents an attractive and less invasive alternative to BM for *MYD88^L265P^* detection [93,94,95,96].

**Figure 3:**
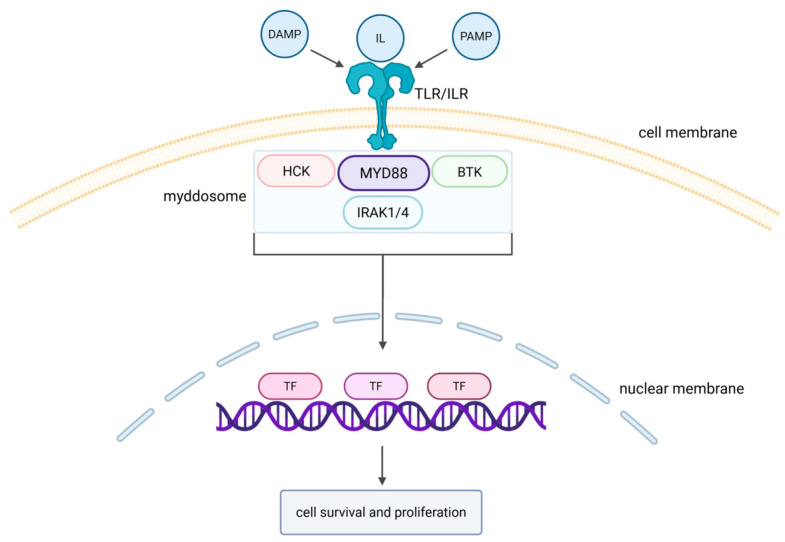
MYD88 signaling pathway in Waldenström macroglobulinemia. Upon stimulation of TLR/ILR by DAMPs, PAMPs, or interleukins, receptor oligomerization leads to assembly of the myddosome complex containing MYD88, IRAK1/4, BTK, and HCK. This activates downstream transcription factors that drive gene expression programs promoting cell survival and proliferation. TF, transcription factor. Created in BioRender. Smith, J. https://app.biorender.com/c-248457.

Functionally, the MYD88 operates as a part of a signaling pathway (Figure 3) that is initiated by the interaction of TLR/IL-1R (interleukin 1 receptor) with DAMPs (damage-associated molecular patterns), PAMPs (pathogen-associated molecular patterns), or interleukins [97,98]. Upon ligand binding, the receptor’s intracellular TIR (Toll/IL-1R) domains oligomerize and recruit the adaptor protein MYD88, which then homodimerizes and activates the kinases IRAK1 (interleukin 1 receptor-associated kinase 1) and IRAK4 (interleukin 1 receptor-associated kinase 4) in a multiprotein complex called the myddosome [97].

In the presence of the *MYD88^L265P^* mutation, the myddosome assembles independently of TLR activation, leading to constitutive activation of IRAKs [99]. This triggers downstream signaling through NFκB, JAK/STAT, and Ras/Erk, ultimately driving increased proliferation and survival of tumor cells [16,81,84,100].

The mutant MYD88 protein can also associate with BTK, a key mediator of BCR signaling, thereby amplifying pathways involved in immune response, cell proliferation, and survival. The MYD88/BTK complex can therefore affect the expression and stability of several oncogenes, e.g., *MYC* or *IL-6* (interleukin 6) [16,101,102]. In addition, *MYD88* mutations enhance activation of HCK (hematopoietic cell kinase), which in turn promotes survival signaling via multiple effectors, including SYK (spleen-associated tyrosine kinase) [103,104,105].

Given its central role in WM pathogenesis, MYD88 has become an attractive therapeutic target. Several approaches targeting this pathway have been proposed in the past, including MYD88 inhibitors [106], MYD88 oligomerization disruptors [107], and gene silencing oligonucleotides [108].

### 4.2. CXCR4

Mutations in the *CXCR4* gene, located on chromosome 2q22.1, are present in approximately 30–40% of WM patients [18]. To date, more than 40 distinct *CXCR4* mutations have been identified in WM. These mutations predominantly affect the C-terminal domain of the protein and are classified as either frameshift or nonsense mutations [84,109].

Frameshift mutations typically disrupt up to 40 amino acids in the C-terminal domain, while nonsense mutations result in premature truncation of the distal 15–20 amino acids [84,109]. The most common variant is the *CXCR4^S338X^*, which introduces a premature stop codon at amino acid position 338, leading to early termination of translation. This mutation arises from a C>A or C>G substitution at position 1013 of the *CXCR4* transcript [109,110].

Mutations in the *CXCR4* C-terminal domain are also found in patients with WHIM syndrome (warts, hypogammaglobulinemia, infection, and myelokathexis), an autosomal dominant immunodeficiency disorder [111]. Acquired *CXCR4* mutations are primarily subclonal and highly associated with coexisting *MYD88* mutations. Notably, individual patients may harbor more than one *CXCR4* mutation [112,113].

Clinically, patients harboring *CXCR4* mutations tend to exhibit distinct disease features. Compared with WT cases, they show less frequent adenopathy but higher rates of BM infiltration, higher serum IgM levels, symptomatic hyperviscosity, and an increased incidence of acquired von Willebrand disease [32,90,110,114,115]. Nonsense mutations, in particular, have been linked to poorer prognosis compared to frameshift mutations [90,115,116].

To understand the biological consequences of these mutations, it is essential to consider the normal role of CXCR4 in hematopoietic cells. CXCR4 is a G-protein-coupled chemokine transmembrane receptor characterized by a conserved motif containing a single amino acid between two cysteine residues. It is expressed on most hematopoietic cell types [117]. In adults, CXCR4 plays a critical role in regulating stem cell retention within the BM [118,119]. Its primary ligand is SDF-1, also known as CXCL12, which is secreted by BM stromal cells [120,121,122].

When the ligand binds to the CXCR4 receptor, signaling pathways that regulate chemotaxis, migration, proliferation, stemness, and the cell cycle are activated. Such signaling pathways include MAPK/Erk and PI3K/Akt [118,123]. Under physiological conditions, CXCR4 signaling is attenuated through phosphorylation of its C-terminal domain by G-protein-coupled receptor kinases GRK2 and GRK3. However, *CXCR4* mutations frequently lead to truncation of this domain, preventing its phosphorylation and thereby sustaining aberrant survival signaling. Notably, higher *CXCR4* expression has also been observed in *CXCR4WT* patients [78].

### 4.3. TP53

p53 plays a crucial role in maintaining genomic stability, regulating the cell cycle, and inducing apoptosis. Abnormalities involving *TP53*, including point mutations and chromosomal deletions, such as loss of the short arm of chromosome 17, are clinically significant events in WM. Reported frequencies of *TP53* mutations (5–25%) and del(17p) (7–15%) vary across studies depending on cohort composition and methodological sensitivity [18,124,125,126,127]. Most *TP53* mutations are missense variants, while frameshift and nonsense mutations occur infrequently [128].

*TP53* mutations are typically subclonal at diagnosis but may expand with the accumulation of genomic instability, reflecting disease evolution and clonal selection [123,124,126]. Although not considered primary drivers of WM initiation, they contribute to aggressive disease biology and are linked to histologic transformation to diffuse large B-cell lymphoma. Therefore, the detection of low-frequency *TP53*-mutated subclones at early disease stages may be clinically relevant for risk stratification and longitudinal monitoring [129,130,131].

Across studies, *TP53* abnormalities correlate with higher BM tumor burden, shorter time to treatment, and inferior clinical outcomes [25,125,128]. They are also associated with treatment resistance and reduced response durability to both chemoimmunotherapy and targeted agents [124,127,132]. Several reports identify *TP53* mutations as an independent adverse prognostic factor for OS and progression-free survival (PFS) [18,25,125,128,132].

Although some smaller studies have not observed statistically significant effects, likely due to cohort heterogeneity, treatment differences, and limited event numbers [126,132,133,134], the overall evidence supports *TP53* as a high-risk molecular feature in WM. Current consensus recommendations advise assessing *TP53* status at diagnosis and prior to each new line of therapy, as these lesions may emerge during disease progression [135].

### 4.4. Other Recurrent Genetic Aberrations

Cytogenetic aberrations are frequently observed in WM, contributing to disease heterogeneity and progression. The most common chromosomal aberration is the deletion of the long arm of chromosome 6 (del(6q)), which occurs in 30–60% of patients. This deletion typically spans the 6q21 to 6q25 region and affects several regulators of key genes like *BTK*, *BCL2,* and *NFκB*, all of which play important roles in B cell survival, apoptosis, and differentiation [110,136,137].

Del(6q) is associated with a more aggressive disease phenotype and a higher likelihood of symptomatic transformation. It generally arises as a secondary event following the acquisition of the *MYD88* mutation, and it rarely co-occurs with *CXCR4* mutations, suggesting distinct evolutionary pathways. This deletion is also commonly found in association with gains in the short arm of chromosome 6, mostly due to the presence of an isochromosome 6, further contributing to genomic complexity [85,136,137,138,139].

Less frequent cytogenetic aberrations in WM include del(13q), del(17p), and trisomy of chromosomes 4, 12, 18 [18,84,125]. As the disease progresses, genomic instability and clonal complexity increase, particularly in relapsed or refractory patients. This is reflected by a higher prevalence of copy number aberrations and other structural chromosomal abnormalities during later stages of the disease [140,141].

**Figure 4:**
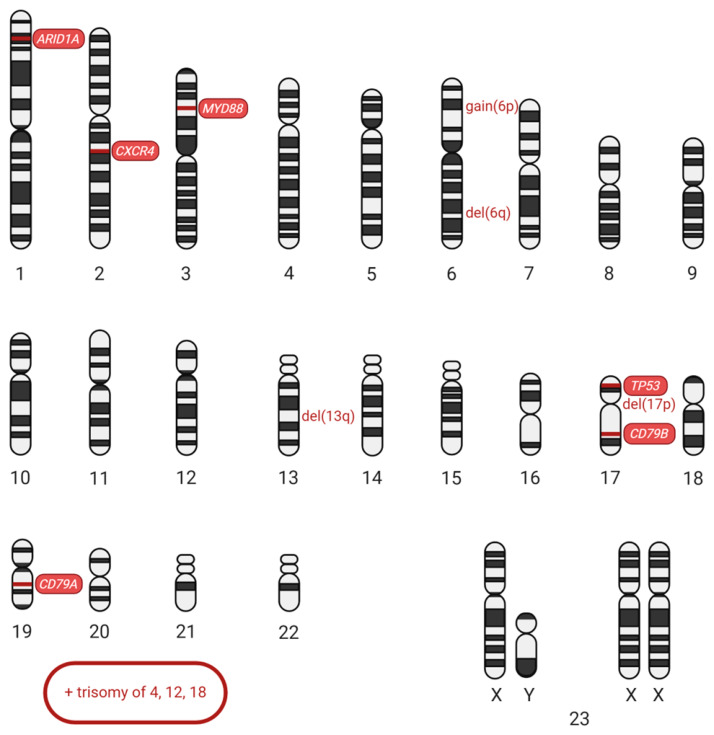
Genetic aberrations commonly found in Waldenström macroglobulinemia and their chromosomal location. Created in BioRender. Smith, J. https://app.biorender.com/c-248457.

In addition, other recurrent somatic mutations have been identified in WM. Point mutations in *ARID1A* (AT-rich interaction domain 1A) are detected in approximately 17% of patients, and mutations in *CD79A* and *CD79B* occur in 8% and 12% of cases, respectively [84,101,124,128,131,134,142]. The most common genetic aberrations identified in WM are depicted in Figure 4.

## 5. Epigenetic Dysregulation

### 5.1. Methylation

Epigenetic alterations, particularly changes in DNA methylation, play a significant role in the pathogenesis and heterogeneity of WM. A study by Roos-Weil et al. investigated *MYD88*-mutated WM patients and stratified them into two subgroups based on their DNA methylation profiles [89]. One group of patients exhibited a methylation pattern resembling that of memory B cells, while the other group showed a profile similar to PCs, which correlates with the results from Gagler et al. [88,89]. A simultaneous analysis of DNA methylation changes in both normal B cell development and WM pathogenesis showed tumor-specific epigenetic events, revealing selective reprogramming of enhancer regions in MBC-like WM and the suppression of heterochromatic regions in PC-like WM. Interestingly, it was observed that *CXCR4* mutations are a defining feature of an earlier MBC-like WM subtype, suggesting a less differentiated, earlier epigenetic stage. In contrast, del(6q) was more frequently observed in the PC-like WM subgroup, indicating a more advanced or differentiated epigenetic state [88].

Further insights into the epigenetic landscape were provided by Chohan et al., who compared the DNA methylation profiles of IgM MGUS patients, WM patients, and healthy controls [71]. When comparing WM/IgM MGUS with healthy controls, a significantly greater number of hypomethylated regions were observed in the 3′UTR, intron, and CpG shores. Conversely, a greater number of hypermethylated regions were observed in the promoter, 5′UTR, exon, and CpG island regions. Comparison of WM to IgM-MGUS showed a significantly greater number of hypomethylated regions in the promoter, 3′UTR, intron, exon, and CpG shore regions. On the other hand, a greater number of hypermethylated differentially methylated regions (DMRs) were seen in the 5′UTR and CpG island regions. These methylation changes correlated with the upregulation of key signaling pathways and genes, including IL-2, IL-6, CXCR4, PI3K/Akt, JAK/STAT, MAPK/Erk, all of which are known to contribute to WM pathobiology [71].

### 5.2. MicroRNA

MicroRNA (miRNA) are short non-coding RNA molecules. CD19+ tumor cells isolated from WM patients display a distinct miRNA expression profile compared to healthy counterparts [143]. Notably, WM cells exhibit upregulation of miR-155-5p, miR-206-3p, miR-494, miR-363, miR-184 and miR-524-3p, alongside downregulation of miR-9-3p and miR-23b-3p. These alterations affect tumor suppressors, cell-cycle regulators, and transcription factors, thereby promoting malignant cell survival and proliferation [144].

Among them, miR-155-5p is particularly important: its inhibition restores cyclin-dependent kinase inhibitors, suppresses MAPK/Erk, PI3K/Akt, and NFκB signaling, reduces *BCL2* expression, and ultimately decreases proliferation, adhesion, and migration of WM cells both in vitro and in vivo [143].

Other dysregulated miRNAs contribute to epigenetic imbalance: miR-206-3p (upregulated) targets histone acetyltransferases, while miR-9-3p (downregulated) targets histone deacetylases, leading to aberrant acetylation and transcriptional dysregulation, and decreased expression of miR-9-3p compared to healthy individuals [144,145]. Reduced expression of miR-23-3p enhances NF-κB activation via SP1 (specificity protein 1) 3′ UTR, which is a transcription factor that positively influences the NFκB pathway [146]. Deletion of 13q removes miR-15a-5p and miR-16-5p, resulting in dysregulated *BCL2* expression and impaired apoptosis [147].

Circulating exosomal miRNAs also exhibit disease-specific changes, with upregulation of miR-192-5p, miR-320b, miR-21-5p, and downregulation of tumor-suppressive let-7d, correlating with disease stage [148,149]. To date, no robust studies have reported other classes of non-coding RNAs in WM.

## 6. Cellular Signaling Networks Beyond MYD88/CXCR4

### 6.1. STAT3

STAT3 is a transcription factor primarily involved in regulating immune responses and inflammation [150]. In B cells, STAT3 activation occurs downstream of cytokine receptors that signal through JAK and TYK kinases, which phosphorylate and activate STAT3, thereby promoting its transcriptional activity. One of the key activating cytokines is interleukin 21 (IL-21) [151]. IL-21 is highly expressed by activated T cells. WM cells express the IL-21 receptor. This interaction induces the secretion and proliferation of IgM in WM cells, primarily through increased expression of BLIMP-1 (a regulator of plasma cell differentiation) and XBP-1 (a regulator of Ig secretion). However, when a STAT3 inhibitor was introduced into the WM cells, this effect was abolished [152].

STAT3 can also be activated indirectly by mutant MYD88, which recruits SYK, a component of the BCR signaling pathway. Inhibition of SYK induces apoptosis in *MYD88*-mutated WM cells, underscoring a mechanistic link between MYD88 signaling and STAT3 activation [104]. Additionally, recent studies have demonstrated significant upregulation of the IL-6/STAT3 pathway in WM, further implicating STAT3 as a central driver of disease biology [80].

### 6.2. FGFR3

Fibroblast growth factor receptors (FGFRs) are tyrosine kinase receptors that mediate the activity of fibroblast growth factors and regulate diverse biological processes, including cell growth, migration, and apoptosis. Among them, FGFR3 has been found to be overexpressed in WM patients compared with healthy controls. FGFR3 phosphorylation activates several downstream pathways, including STAT3, Raf, and Erk [153]. Pharmacologic inhibition of FGFR signaling with tyrosine kinase inhibitors has been shown to effectively silence these pathways in WM cells [153].

More recently, Sacco et al. demonstrated that FGF traps, which bind FGFs, can inhibit WM cell proliferation and induce apoptosis. Blockade of FGF/FGFR signaling also suppressed MYD88-driven pathways, including BTK, SYK, and HCK, highlighting crosstalk between FGFR and canonical WM signaling pathways [154].

### 6.3. PI3K

PI3K is a central component of the PI3K/Akt/mTOR signaling pathway, which regulates cell proliferation, survival, immunity, metabolism, and lymphocyte trafficking [155,156]. PI3K can be activated through multiple receptors, including FGFR, BCR, and TLR [155]. Upon activation, PI3K phosphorylates phosphatidylinositol-4,5-bisphosphate (PIP2) to generate phosphatidylinositol-3,4,5-trisphosphate (PIP3), which recruits oncogenic signaling proteins, such as Akt. Akt in turn phosphorylates several substrates, including the mTOR protein kinase, typically functioning within larger complexes [155]. The phosphatase PTEN (phosphatase and tensin homolog) serves as a crucial negative regulator of the PI3K/Akt/mTOR axis [155].

In the context of WM, hyperactivation of Akt has been consistently described. Leleu et al. reported higher levels of phosphorylated Akt in CD19+ BM cells from WM patients compared with CD19+ BM cells from healthy donors. They also described baseline Akt phosphorylation in WM cell lines [156]. Similarly, Roccaro et al. found that PTEN expression was significantly reduced in CD19+ WM cells compared to normal CD19+ control cells, resulting in constitutive activation of the PI3K/Akt pathway [157]. Pharmacologic inhibition of PI3K, Akt, or mTOR has been shown to induce cytotoxicity and apoptosis in WM cells [156,157,158,159,160].

### 6.4. IL-6

IL-6 is a proinflammatory cytokine with physiological roles in B cell differentiation and Ig production [161]. It has been shown that serum levels of IL-6 and its receptor IL-6R are significantly elevated in WM patients, suggesting a pathogenic role in this malignancy [162]. In WM cells, IL-6 promotes IgM production through CCL5 (C-C motif chemokine ligand 5) signaling. CCL5 concentrations are markedly increased in both serum and BM of WM patients, where they positively correlate with disease aggressiveness [163]. CCL5 then induces IL-6 expression in WM stromal cells via the GLI2 transcription factor and the PI3K/Akt pathway [163,164].

Subsequently, IL-6 activates the JAK/STAT pathway in WM cells, leading to enhanced IgM secretion [163]. Additionally, GLI2 has been identified as a transcriptional regulator of IL-6R expression in WM cells [165]. Therapeutic targeting of either GLI2 or IL-6R was shown to reduce IgM expression by WM cells, underscoring their importance in WM pathogenesis [165,166].

## 7. Therapy

### 7.1. Therapy of Today

The primary goal of treatment is to achieve long-term symptom control and maintain quality of life. Therapy should be administered only to those patients who are symptomatic [15,167]. The level of monoclonal IgM alone is not an indication to initiate treatment [168,169]; however, among patients with high IgM levels (>6000 mg/dL), data are conflicting [170,171]. The indicative symptoms of treatment include hyperviscosity, neuropathy, symptomatic adenopathy or organomegaly, amyloidosis, cryoglobulinemia, cold agglutinin disease, anemia, B symptoms, and cytopenia [109,167,168,172]. For patients requiring immediate disease control, such as those with symptomatic hyperviscosity, initial plasmapheresis is recommended. After plasmapheresis, systemic treatment should be initiated as soon as possible [173].

Since WM is a rare disease, only a few randomized trials and limited data are available for comparing different treatment approaches. No single optimal treatment regimen has yet been established; therefore, the choice of treatment must consider the patient’s current clinical status (clinical symptoms, tumor burden, requirement for rapid response) and mutational/genomic status. Assessing *MYD88*, *CXCR4,* and *TP53* mutational status is an emerging approach for genomically driven therapy [174].

A subset of symptomatic WM patients displays unfavorable outcomes. The most recent expert recommendation from the 12th International Workshop on Waldenstrom’s Macroglobulinemia defined high-risk symptomatic disease as a combination of: advanced chronological age, low serum albumin, elevated serum lactate dehydrogenase, elevated beta-2-microglobulin (β2M), and the presence of *TP53* alterations. Routine assessment of *TP53* mutations and del(17p) is now recommended before starting a new line of therapy [135].

Relapse is common in WM. The choice of subsequent therapy depends on prior treatment, its tolerability, and the duration of response [175,176]. Additional factors that influence treatment selection are: mutational status (*MYD88*, *CXCR4*, *TP53*), biological age, comorbidities, transplant eligibility, and patient preference [175]. Retreatment with the same regimen may be appropriate after a durable remission, while early relapse (<24 months) or refractory disease requires a switch to an alternative class of drugs [15].

Given the increasing number of available therapeutic options, current treatment approaches in WM are summarized in Table 2 to provide a concise overview of their clinical use, advantages, and limitations.

### 7.2. Future Directions

In efforts to enhance the efficacy of chemoimmunotherapy and to explore time-limited BTKi strategies, several studies evaluate combinations with proteasome inhibitors: (ECWM-1 trial: DRC with bortezomib, FIL-BRB study: BR + bortezomib) or BTKi (BRAWN trial: BR with acalabrutinib; ZBR trial: BR with zanubrutinib) [206,207,208,209]. The ECWM-1 study in newly diagnosed symptomatic WM demonstrated that adding bortezomib to DRC increased response depth without compromising safety, although no PFS benefit was observed [183]. In the prospective FIL-BRB study of relapsed WM, BR + bortezomib achieved a high response rate and prolonged PFS in indirect comparison with BR alone (30-month PFS of 79%; vs. 2-year PFS of 66%) [207,210]. In ZBR trial for treatment-naïve WM, six cycles of BR with zanubrutinib given for one year resulted in a 100% response rate with 93% major responses a 57% MRD negativity [209].

Targeted agents, such as antibody-drug conjugates, BCL2 inhibitors, phospholipid radioconjugates, noncovalent BTKi, and bispecific antibodies, represent the modern direction of WM therapy. There are ongoing studies exploring agents, such as loncastuximab teserine (NCT05190705), sonrotoclax as monotherapy and in combination with zanubrutinib (NCT05952037), and ipofosine (NCT02952508), as well as combinations including carfilzomib with ibrutinib (NCT04263480), pirtobrutinib with venetoclax (NCT05734495), and rituximab with pembrolizumab (NCT03630042).

Novel investigational compounds include bifunctional proteolysis-targeting chimeras (PROTACs) and ubiquitin-based molecular degraders designed to eliminate key components of the BCR pathway in *MYD88*-mutated WM [211,212,213].

Cellular therapy with CAR-T cells has also entered WM research. In a retrospective analysis of patients with WM heavily pretreated prior to CAR-T, commercial anti-CD19 products (axi-cel and tisa-cel) achieved a 95% ORR, including 86% complete remissions, with 12-month PFS and OS of 70% and 84%, respectively. It is important to mention that these specific results were observed in histologically transformed WM, so the outcomes might differ significantly for non-transformed, indolent WM [214].

## 8. Conclusions

WM is a rare B-cell malignancy characterized by the clonal expansion of lymphoplasmacytic cells, overproduction of monoclonal IgM, and a heterogeneous clinical presentation. Over the past decade, major progress has been made in unraveling its molecular basis. The discovery of recurrent *MYD88^L265P^* and *CXCR4* mutations has provided key diagnostic and prognostic markers. Studies on intracellular signaling (NF-κB, PI3K/Akt/mTOR, JAK/STAT, BCR-associated kinases) and microenvironmental interactions have deepened our understanding of disease biology.

Therapeutically, BTKi have transformed the treatment paradigm, achieving durable responses in many patients. Nevertheless, resistance, particularly in *MYD88WT*, *CXCR4*-mutated, and *TP53*-altered disease, remains a major obstacle. New approaches, such as BCL2 inhibitors, PI3K/mTOR pathway blockade, noncovalent BTKi, antibody–drug conjugates, bispecific antibodies, and cellular adoptive immunotherapy, provide additional options but are not yet curative. Genomically driven therapy based on *MYD88*, *CXCR4*, and *TP53* status is a current direction of WM care. Emerging research directions are further expanding the field. Epigenetic reprogramming and miRNA dysregulation have been identified as important contributors to WM pathogenesis and hold promise as biomarkers for disease monitoring and therapeutic stratification. Moreover, the identification of circulating miRNAs and methylation signatures may facilitate minimally invasive diagnostics and guide precision medicine strategies.

Looking forward, the greatest challenges will be overcoming resistance to BTKi, developing effective drug combinations, and harnessing novel immunotherapies. Integrating genomic, epigenomic, and immune profiling into clinical decision-making will be crucial for personalizing therapy and enhancing patient outcomes. Ultimately, translating these molecular insights into rational therapeutic strategies offers the best path toward durable disease control and potentially cure of WM.

## Figures and Tables

**Table 1:** Molecular clusters identified in IgM monoclonal gammopathies based on transcriptional, epigenetic, metabolic, and immune features.

Cluster	Patient Composition	Main Molecular Features	Immune Microenvironment	Clinical Association
1	Exclusively WM patients	Mitochondrial metabolism; transcriptional silencing of cell-cycle and immune-response pathways; *TNFAIP3* alterations	Infiltration by senescent T effector memory cells	Aggressive clinical course
2	Both IgM MGUS and WM patients	Inflammatory response, senescence, and glycolysis	Increased activated T follicular helper cells and regulatory T cells	Indolent clinical course
3	Both IgM MGUS and WM patients	Intermediate proliferative, inflammatory, glycolytic, and mitochondrial profile	Intermediate immune-related features	Intermediate or less clearly defined clinical behavior

WM, Waldenström macroglobulinemia; MGUS, monoclonal gammopathy of undetermined significance; *TNFAIP3*, tumor necrosis factor alpha-induced protein 3.

**Table 2:** Current therapeutic approaches in Waldenström macroglobulinemia.

Therapeutic Approach	Examples/ Regimens	Preferred Clinical Context	Main Advantages	Key Limitations/Considerations	References
Plasmapheresis	Plasmapheresis followed by systemic therapy	Symptomatic hyperviscosity or need for immediate IgM reduction	Rapid reduction in circulating IgM and viscosity-related symptoms	Does not control the underlying disease; systemic therapy should follow as soon as possible	[173]
Rituximab-based chemoimmunotherapy	BR; DRC	First-line treatment of symptomatic WM; retreatment may be considered after durable prior response	Time-limited treatment; BR provides deeper and more durable responses than DRC	DRC is less intensive but associated with lower response depth and shorter PFS; rituximab monotherapy has limited activity and slow responses	[177,178,179,180,181,182,183]
Proteasome inhibitor-based regimens	BDR; CRD; ixazomib-rituximab-dexamethasone	Frontline or relapsed setting; particularly useful in WM-related AL amyloidosis or light chain deposition disease	Rapid activity; ixazomib offers an oral option; carfilzomib avoids bortezomib-associated neurotoxicity	Bortezomib may cause peripheral neuropathy; carfilzomib carries cardiac and pulmonary toxicity risks, especially in older patients	[15,183,184,185,186,187,188,189]
Covalent BTKi	Ibrutinib ± rituximab; zanubrutinib; acalabrutinib; tirabrutinib; orelabrutinib	Standard first-line option or relapsed WM, especially after chemoimmunotherapy	Highly effective targeted therapy; zanubrutinib has improved safety compared with ibrutinib and is generally favored in *CXCR4*-mutated, *TP53*-altered, or *MYD88WT* disease	Continuous treatment; ibrutinib is associated with atrial fibrillation, hypertension, and bleeding; intolerance may require dose reduction or switching	[83,135,174,190,191,192,193,194,195,196,197]
Non-covalent BTKi	Pirtobrutinib	Relapsed/refractory WM, particularly after prior covalent BTKi exposure	Activity after covalent BTKi therapy; targets both wild-type and mutated BTK	Optimal sequencing remains to be defined	[198,199]
BCL2 inhibition	Venetoclax; sonrotoclax under investigation	Relapsed WM, including patients previously exposed to BTKi and those with *CXCR4* mutations	Targeted, chemotherapy-free approach; high response rates reported in the relapsed setting	Optimal combinations, duration, and sequencing remain under investigation	[200,201]
Alternative/less commonly used agents	Cladribine; everolimus; ofatumumab; immunomodulatory drugs	Selected relapsed/refractory cases when preferred options are unsuitable	May provide disease control in selected patients	Limited role due to modest efficacy or toxicity	[185,202,203,204,205]
Hematopoietic cell transplantation	Autologous or allogeneic transplantation in selected cases	Selected patients with relapsed/refractory disease, preferably after BTKi failure	Potential option for heavily pretreated or high-risk patients	Applicable only to carefully selected patients; toxicity and patient fitness must be considered	[172]

WM, Waldenström macroglobulinemia; IgM, immunoglobulin M; BR, bendamustine-rituximab; DRC, dexamethasone-rituximab-cyclophosphamide; BDR, bortezomib-dexamethasone-rituximab; CRD, carfilzomib-rituximab-dexamethasone; BTKi, Bruton tyrosine kinase inhibitor; PFS, progression-free survival.

## Data Availability

No new data were created or analyzed in this study. Data sharing is not applicable to this article.

## Abbreviations

The following abbreviations are used in this manuscript:

Akt	protein kinase B
ARID1A	AT-rich interaction domain 1A
ATM	ataxia telangiectasia mutated
anti-MAG	anti-myelin-associated glycoprotein
β2M	beta-2-microglobulin
BCR	B cell receptor
BDR	bortezomib, dexamethasone, rituximab
BLIMP-1	regulator of plasma cell differentiation
BM	bone marrow
BR	bendamustine, rituximab
BTK	Bruton’s tyrosine kinase
BTKi	Bruton’s tyrosine kinase inhibitors
CCL5	C-C motif chemokine ligand 5
CLL	chronic lymphocytic leukemia
CRD	carfilzomib, rituximab, dexamethasone
ctDNA	circulating tumor DNA
CXCL12	C-X-C chemokine ligand 12
CXCR4	C-X-C chemokine receptor 4
DAMPs	damage-associated molecular patterns
ddPCR	droplet digital PCR
DMRs	differentially methylated regions
DRC	dexamethasone, rituximab, cyclophosphamide
Eph-B2	ephrin receptor B2
FGFRs	fibroblast growth factor receptors
HCK	hematopoietic cell kinase
IGH	immunoglobulin heavy locus
IgM	monoclonal immunoglobulin M
IgM MGUS-NOS	IgM MGUS-not otherwise described
IgM MGUS-PC	IgM MGUS-plasma cell
IL-1R	Interleukin 1 receptor
IL-6	interleukin 6
IL-21	interleukin 21
IRAK1	interleukin 1 receptor-associated kinase 1
IRAK4	interleukin 1 receptor-associated kinase 4
JAK	Janus kinase
MBC-like	memory B cell-like
MGUS	monoclonal gammopathy of undetermined significance
MM	multiple myeloma
MRD	minimal residual disease
MYD88	myeloid differentiation primary response 88
NF-κB	nuclear factor kappa light chain enhancer of activated B cells
OS	overall survival
PAMPs	pathogen-associated molecular patterns
PC	plasma cell
PIP2	phosphatidylinositol-4,5-bisphosphate
PIP3	phosphatidylinositol-3,4,5-trisphosphate
PI3K	phosphatidylinositol 3-kinase
PROTACs	proteolysis-targeting chimeras
PTEN	phosphatase and tensin homolog
SDF1	stromal cell-derived factor 1
STAT	signal transducer and activator of transcription proteins
SYK	spleen associated tyrosine kinase
TIR	toll/IL-1R
TLR	toll-like receptor
Tregs	T regulatory cells
TNFAIP3	tumor necrosis factor alpha-induced protein 3
WM	Waldenström macroglobulinemia
wt	wild-type
XBP-1	regulator of Ig secretion
